# Lipid Priming of Adipose Mesenchymal Stromal Cells with Docosahexaenoic Acid: Impact on Cell Differentiation, Senescence and the Secretome Neuroregulatory Profile

**DOI:** 10.1007/s13770-024-00679-5

**Published:** 2024-11-04

**Authors:** Jonas Campos, Belém Sampaio-Marques, Diogo Santos, Sandra Barata-Antunes, Miguel Ribeiro, Sofia C. Serra, Tiffany S. Pinho, João Canto-Gomes, Ana Marote, Margarida Cortez, Nuno A. Silva, Adina T. Michael-Titus, António J. Salgado

**Affiliations:** 1 Life and Health Sciences Research Institute (ICVS), School of Medicine, University of Minho, 4710-057 Braga, Portugal; 2 ICVS/3B’s – PT Government Associate Laboratory, Braga/Guimarães, Portugal; 3 Centre for Neuroscience, Surgery and Trauma, The Blizard Institute, Barts and The London School of Medicine and Dentistry, Queen Mary University of London, London, E1 2AT UK

**Keywords:** Mesenchymal stem cell, Secretome, Docosahexaenoic acid, Senescence, Neural differentiation

## Abstract

**Background:**

Priming strategies that improve the functionality of MSCs may be required to address issues limiting successful clinical translation of MSC therapies. For conditions requiring high trophic support such as brain and spinal cord injuries, priming MSCs to produce higher levels of trophic factors may be instrumental to facilitate translation of current MSC therapies. We developed and tested a novel molecular priming paradigm using docosahexaenoic acid (DHA) to prime adipose tissue-derived mesenchymal stromal cells (ASCs) to enhance the secretome neuroregulatory potential.

**Methods:**

Comprehensive dose–response and time-course assays were carried to determine an optimal priming protocol. Secretome total protein measurements were taken in association with cell viability, density and morphometric assessments. Cell identity and differentiation capacity were studied by flow cytometry and lineage-specific markers. Cell growth was assessed by trypan-blue exclusion and senescence was probed over time using SA-β-gal, morphometry and gene expression. Secretomes were tested for their ability to support differentiation and neurite outgrowth of human neural progenitor cells (hNPCs). Neuroregulatory proteins in the secretome were identified using multiplex membrane arrays.

**Results:**

Priming with 40 µM DHA for 72 h significantly enhanced the biosynthetic capacity of ASCs, producing a secretome with higher protein levels and increased metabolic viability. DHA priming enhanced ASCs adipogenic differentiation and adapted their responses to replicative senescence induction. Furthermore, priming increased concentrations of neurotrophic factors in the secretome promoting neurite outgrowth and modulating the differentiation of hNPCs.

**Conclusions:**

These results provide proof-of-concept evidence that DHA priming is a viable strategy to improve the neuroregulatory profile of ASCs.

**Supplementary Information:**

The online version contains supplementary material available at 10.1007/s13770-024-00679-5.

## Introduction

Mesenchymal stromal cells (MSCs) constitute an established cell source for development of therapeutic applications for several diseases. MSCs can be generally defined as having a mesenchymal origin, being first isolated from the bone-marrow [1] and later from several body compartments such as, adipose tissue and umbilical cord among others [2]. Their high proliferative capacity, ability to adhere to plastic and sustain fibroblastoid morphology in culture are some of their defining characteristics. Specific criteria have been proposed in order to phenotypically and functionally define these cells. Respectively, MSCs require positive expression of tetraspanins such as (CD73, CD90, CD105 and CD44) and negative expression of hematopoietic markers (CD45 and HLA-DR) combined with the capacity to differentiate along the osteogenic, adipogenic and chondrogenic lineages [3]. Together, these criteria conceptually ensure that these cells hold therapeutic potential [4]. Due to its relative pathophysiological complexity, central nervous system (CNS) disorders are viable candidates where MSCs could exert therapeutic benefits [5]. The pleiotropic mechanisms related to MSCs functionality for therapeutic applications are now established to be derived mostly from their paracrine activity [5]. The MSC secretome, defined by the combination of secreted cytokines, growth-factors, bioactive lipids, metabolites and extracellular vesicles have been employed in several pre-clinical trials of CNS disorders such as Parkinson’s disease [6–9] and spinal cord injury [10, 11]. Although these initial experimental studies demonstrate, solid proof-of-concept evidence for the effectiveness of the MSCs secretome, the move towards clinical translation may require secretomes with higher and more tailored therapeutic activity [12]. In a recent International Society for Cell and Gene Therapy (ISCT) publication, the use of priming strategies to induce positive phenotypic alterations that lead to increased MSCs basal fitness was touted as a key option [13]. Several priming strategies have been studied as a way to induce beneficial phenotypic changes in MSCs leading to (higher trophic support and immune modulation) for improved therapeutic potential [14]. While some studies focus on the manipulation of culture conditions such as changes in oxygen tension [15] or bioreactor 3D culturing [16], the majority adopts pro-inflammatory stimuli, involving the use of IFN-γ alone or in combination with TNF-α or IL1-β [14]. In this study we aimed to employ a strategy inspired by the biology of the adipose tissue, to prime human adipose-derived mesenchymal stromal cells (ASCs). In the adipose tissue, ASCs are located in the stroma-vascular fraction, specifically positioned at or near to the vasculature, where they are able to sense their microenvironment, coupling nutritional and metabolic fluxes to determine adipogenic fate commitment [17, 18]. ASCs sense these metabolic cues using specialized signaling organelles called primary cilia, through the presence of the free-fatty acid receptor 4 (FFAR4) [19]. Docosahexaenoic acid (DHA), a natural FFAR4 ligand, has been shown to drive beneficial responses in adipose progenitor cells leading to adipogenic transcriptional changes and the production of anti-inflammatory cytokines [18, 20, 21]. Taking advantage of this interaction, we studied the effects of DHA priming on ASCs biology using several experimental paradigms. Furthermore, we then developed a DHA priming strategy to expand and collect ASC secretome with an improved neuroregulatory profile for future CNS applications. Our results demonstrated that DHA-primed ASCs have increased metabolic activity and biosynthetic capacity which combined led to the production of secretome with higher protein concentration. These phenotypic changes were translated into a higher rate of adipogenic differentiation and attenuated senescence phenotype after multiple passages. As a proof-of-concept, we demonstrated that this DHA-primed secretome was superior to the traditional ASCs secretome in inducing neurite outgrowth and neural differentiation using an established *in vitro* assay.

## Materials and methods

### Cell culture

Human adipose tissue derived mesenchymal stromal cells (ASCs, lot:080313), kindly provided by Professor Jeffery Gimble (Obatala Sciences LLC, New Orleans, LA, USA) were cultured according to previously established protocols in α-MEM media (Invitrogen, Waltham, MA, USA) supplemented with 10% FBS (S0615, Sigma-Aldrich, St. Louis, MO, USA) and 1% antibiotic–antimycotic mixture (Invitrogen) [22]. For further reagent information, refer to Supplementary Table 3.

## DHA treatments and secretome collection

Neat docosahexaenoic acid (DHA) (D2534, Sigma-Aldrich) was aliquoted as a stock solution (100 mM) in ethanol followed by nitrogen flushing and kept at −80 °C until further use. DHA concentrations (1, 10, 25 or 40 µM) were prepared and complexed with fatty acid free bovine serum albumin (FFA-BSA) (A8806, Sigma-Aldrich) at 1% of the total media. Controls were cells kept in media without any DHA or vehicle (Control) and cells exposed only to the FFA-BSA and ethanol vehicle (Veh Control). The treatment of P6 cells started always 48 h after plating (4 × 10^3^) cells/cm^2^ and was maintained for up to 72 h. Medias were then removed and cells were washed five times with PBS without Ca2^+^ and Mg2^+^ (Invitrogen) and fresh Neurobasal-A media (TermoFisher Scientific, Waltham, MA, USA) with 1% kanamycin (Life Technologies, Carlsbad, CA, USA) was added for secretome production. Secretomes from treated (ω-SEC) and non-treated cells (SEC) were harvested after 24 h, and centrifuged at 1000 g for 10 min 4 °C to remove cell debris, followed by snap-freezing in liquid nitrogen and storage at −80 °C until use.

## Assessment of cellular density and morphology

At 24, 48 and 72 h after treatment with different DHA concentrations, cells were fixed with 4% paraformaldehyde (PFA; PanReac, Castellar del Vallès, Barcelona, Spain) for 15 min at room temperature (RT°) followed by two 5 min PBS washes. Subsequently, TRITC-complexed phalloidin 0.1 μg/mL (Sigma-Aldrich) and DAPI 0.1 μg/mL (Invitrogen) were added to the cells in PBS with 0.1% Triton X-100 for 45 min followed by three PBS washes for 5 min, to reveal the actin cytoskeleton and nucleus respectively. Fluorescence images were acquired using a motorized wide-field IX81 inverted microscope (Olympus, Shinjuku, Tokyo, Japan) equipped with a 20 × objective (Microscope Objective LUCPLFLN NA 0.45). Four images per well of a 24 multi-well plate were acquired using random sampling with multi-point acquisition. In total, 12 wells per group across three individual assays were imaged using the same acquisition parameters. Image analysis was carried out using Fiji (Baltimore, MD, USA) [23]. Briefly, to obtain quantifications and morphometric information of cell nuclei, the DAPI signal was automatically segmented using global Otsu thresholding and quantified using the analyze particles plug-in with a minimum size filter of 25 µm. The phalloidin signal was invert-segmented based on the background signal followed by the subtraction of the background area from the total image area. The total cytoplasm area retrieved from each image was then divided by the number of counted cells to obtain an average cell size. The temporal evolution of these morphometric parameters can be found in (Supplementary Fig. 1).

## Assessment of metabolic viability

Following 24, 48 and 72 h of treatment with different DHA concentrations, media was removed, and cells were washed once in PBS followed by a 3 h incubation with a 1:5 dilution of MTS reagent (CellTiter 96®, Promega, Madison, WI, USA) in DMEM (Sigma) without FBS. Afterwards, the media was collected and read in three technical replicates per well using a microplate reader (Infinite® 200PRO, Tecan, Männedorf, Zurich, Switzerland) at 490 nm.

## Secretome total protein evaluation

Protein measurements were performed using an adjusted micro-Bradford assay (Quick Start™, BioRad, Hercules, CA, USA). Briefly, a 10-point standard curve was built in triplicate using a BSA standard ranging from 0 to 50 µg/mL of protein. One-hundred and fifty microliters of secretome samples from each DHA treatment concentration and respective controls were loaded in duplicate into 96 flat multi-well plates, followed by the addition of 150 µL of Bradford reagent and incubation at RT° for 10 min protected from light. Plates were read using a microplate reader (Infinite® 200PRO, Tecan) at 595 nm. Absorbance values were converted to concentration using the standard curve.

## Cell phenotyping

For flow cytometry studies, P6 cells were treated with 40 µM DHA for 72 h without media change. At 80–90% confluence, cells were washed five times in PBS and conditioned as for secretome collection. After 24 h, cells were detached from the wells using 0.05% trypsin–EDTA and counted using the trypan blue exclusion assay (Invitrogen) in a Neubauer chamber (Marienfield, Lauda-Königshofen, Stuttgart, Germany). Then, 1 × 10^5^ cells were transferred to a round bottom 96-well plate well and resuspended in PBS. Cells were then incubated for 20 min at 4ºC with the following antibodies: PE anti-human CD90 (clone 5E10), PE/Cy7 anti-human CD73 (clone AD2), FITC anti-human CD105 (clone 43A3), PerCP-Cy5.5 anti-human CD44 (clone IM7) Brilliant Violet 605™ anti-human CD45 (clone HI30) and Brilliant Violet 510™ anti-human HLA-DR (clone L243) (all from BioLegend, San Diego, CA, USA). Cells were then washed and re-suspended in PBS buffer. All samples were acquired on an eight-colour BD LSRII flow cytometer using the FACS DIVA software version 6.0 (Becton Dickinson, USA). Data, represented as percentage of positive cells for each marker, was analyzed with the FlowJo software version 10.0.8 (Becton Dickinson, Franklin Lakes, NJ, USA) as described in (Supplementary Fig. 2).

## Multi-lineage differentiation

Cells seeded at P6 in 24 multi-well plates were treated with 40 µM DHA for 72 h followed by the normal conditioning protocol for secretome production. Following 24 h, cells reach full confluence and are ready to initiate the differentiation protocols. Differentiation along the osteogenic, adipogenic and chondrogenic lineages was carried out using commercially available kits (STEMCELL Technologies, Vancouver, BC, Canada) and according to manufacturer’s instructions. Briefly, cells are cultured in the provided supplemented medias for 14 days for osteogenic and adipogenic, and for 21 days for chondrogenic differentiation with media changes every 72 h. Chondrogenic differentiation was performed as a pellet, with initial plating density of 5 × 10^5^ cells in round bottom 15 mL tubes. To maintain correct pellet morphology and hypertrophy the tubes were flicked at each media change. Successful differentiation was confirmed by robust pellet hypertrophy and accumulation of proteoglycan along the borders of the pellet. At the end of the differentiation time-points, cells were fixed for 15 min in 4% (PFA; PanReac) and washed with PBS. The resulting pellet from chondrogenic differentiation was fixed in 4% PFA for 30 min, washed with PBS and embedded in Tissue-Tek® O.C.T.™ compound (Sakura Finetek, Chuo-ku, Tokyo, Japan), gradually frozen in liquid nitrogen, sequentially sectioned at 20 μm thickness in a cryostat (CM1900, Leica, Wetzlar, Hesse, Germany) and finally collected onto SuperFrost® Plus slides (ThermoFisher Scientific). For immunofluorescence staining, samples were permeabilized and blocked for 45 min in PBS 0.3% Triton X-100 (0.3% PBS-T), and 10% newborn calf serum (NBCS, ThermoFisher Scientific). Then, primary antibodies were added in the same medium but with 4% NBCS at RT° for 4 h: goat anti-FABP4 for adipogenic, mouse anti-Osteocalcin for osteogenic or goat anti-Aggrecan for the chondrogenic differentiation, all at 10 µg/mL (R&D Systems, Minneapolis, MN, USA). Subsequently, the samples were washed with PBS, and incubated at RT° with the secondary antibodies for 2 h in the same incubation medium: Alexa Fluor 488 goat anti-mouse or Alexa Fluor 488 rabbit anti-goat (1:1000, Invitrogen). Cells were washed with PBS, and finally, a counterstaining with DAPI (1 μg/mL, Invitrogen) was performed for 10 min at RT°. SuperFrost slides were mounted using Permafluor (Thermo Fisher Scientific) and images were acquired at 20 × magnification using the IX81 fluorescence microscope (Olympus). Differentiation along the osteogenic and adipogenic lineages was confirmed by assessing specific marker expression together with lineage-dependent morphological adaptations such as the presence of cobblestone-like morphology and growth of lipid filled vacuoles respectively. Analysis of the adipogenic phenotype was carried in Fiji, using the polygon tool to manually segment cells from 10 images/well across 4 individual wells per group. After segmentation of adipocyte cytoplasm, the number and areas occupied by lipid vacuoles were measured based on the automatic threshold of the inverted signal of FABP4 staining. Quantification was carried out using the analyze particles plug-in with a size filter ranging from 2 to 100 µm^2^ and a circularity filter ranging from 0.55 to 1.00.

## Growth dynamics throughout secretome production and continuous passages

To study cell growth throughout the secretome production pipeline, P6 cells were plated and treated with 40 µM DHA as previously. Daily cell samples (from treatment baseline to 120 h post treatment) were collected after 0.05% trypsin–EDTA detachment and counted in triplicates using the trypan blue exclusion assay (Gibco, Grand Island, NY, USA). To assess population doubling time along multiple passages (P4 to P13), cells were harvested and quantified only at 90% confluence as previously established [24]. Briefly, cumulative population doublings (cPD) were calculated for each condition from P4 onwards using the following formula:

$$cPD = cPD(0)+3.322 * (log(Nf) -log(Ni))$$

where cPD(0) is the cPD of the previous passage, Ni is the number of seeded cells and Nf is the number of cells counted at the end of the passage. An outline of the experimental procedures of both paradigms is provided in (Supplementary Fig. 3).

## Assessment of replicative senescence associated markers

Cells expanded from P4 to P13 were probed at specific passages with a staining kit for SA-β-galactosidase activity to examine the induction of replicative senescence (Supplementary Fig. 3A). Briefly, cells were plated in 6-well plates and treated with DHA 40 µM for 72 h. After reaching 80–90% confluence, cells were washed with PBS and fixed with the kit fixative solution for 10 min. Cells were then washed with PBS and 2 mL of staining solution, containing 20 mg/mL of 5-bromo-4-chloro-3 indolyl-β-D-galactopyranoside (X-Gal), was added to each well. The plate was sealed with parafilm, and the cells were incubated ON at 37ºC. The next day, after nuclei counterstaining with DAPI for 10 min at RT°, ten bright-field images per well and the corresponding fluorescence images were acquired with a IX53 wide-field inverted microscope (Olympus). To evaluate morphological changes, cells at the same passages were stained with phalloidin and DAPI as previously, and imaged at 10 × magnification (UPLFLN, NA 0.3). For cell area quantification, the phalloidin signal was segmented with a minimal error algorithm threshold followed by outlier removal of bright and dark pixels with settings of radius = 1 and threshold = 50. Areas from each image were normalized to the total cell numbers quantified from the DAPI signal to provide single cell area data in square micrometers. SA-β-galactosidase areas were measured using color-thresholding of the RGB space set for a hue of blue, and were normalized by the number of cells wihtin each image. 

## Senescence associated gene expression analysis

At P6 and P12, cells were seeded into 6-well plate wells, as previously described. Upon confluence of 80–90%, total RNA was extracted using TripleXtractor (Grisp, Portugal). RNA yield and purity was analyzed using a NanoDrop 1000 spectrophotometer (ThermoFisher Scientifc) presenting (> 1.8—260/280) ratios. One microgram of RNA from each sample was converted to cDNA using the Xpert cDNA Supermix with gDNA eraser (Grisp, Porto, Portugal). Primers were designed using Primer-BLAST (NCBI, United States) according to the respective GenBank sequence and individually tested for reaction efficiency. The names of the genes and the respective primers are described in Supplementary Table 1. The qRT-PCR was performed using equal cDNA concentrations for each condition in a 7500 Fast RealTime PCR System (ThermoFisher Scientifc) with the XPert Fast SYBR mastermix (Grisp) according to manufacturer’s instructions. Melting curves exhibited a single sharp peak at the expected temperature. Expression of target genes (P16^INK4A^, P21^CIP1^ and P53) was normalized to the geometrical mean expression of the housekeeping genes (GAPDH, PPIA and B2M) using the 2^−ΔΔCt^ method.

## Multiplexed detection of neuroregulatory proteins

To evaluate how DHA treatment modulated the secretion of different chemokines, cytokines and trophic factors with important neuroregulatory functions, a membrane-based antibody array (Human Neuro Discovery Array C1, AAH-NEU-1–2, RayBiotech, Peachtree Corners, GA, USA) was used, according to manufacturer’s instructions. Briefly, after 30 min of blocking, the membrane array was incubated overnight, at 4ºC with secretomes (4 samples/group) with matched total protein concentrations. The next day, the membrane was incubated with the biotinylated detection antibody cocktail, followed by incubation with streptavidin conjugated HRP. Chemiluminescence detection was performed using Sapphire Biomolecular Imager and spot intensity was measured using AzureSpot software (Azure Biosystems, Dublin, CA, USA).

## Human neural progenitor cell cultures

hNPCs were kindly provided by Prof. Leo A. Behie (University of Calgary, Canada). Cells were isolated from the telencephalic region of a 10-week post-conception (gestational age) fetus, within ethical guidelines previously established and approved by the Conjoint Health Research Ethics Board (CHREB, University of Calgary, Canada; ID: E-18786) as described previously [16]. Cryopreserved hNPCs were thawed at 37 °C and placed into a T-25 flask containing 5 mL of Complete NeuroCult™ Proliferation Medium (Stem Cell Technologies). After 2 days, the cells were harvested and mechanically dissociated into a single cell suspension and subcultured in fresh media. Every 2 days, 40% of the medium was replaced with fresh Complete NeuroCult™ Proliferation Media. Neurospheres were passaged after 7–14 days, according to the protocol described by the manufacturer. Briefly, neurospheres were harvested from the flask, centrifuged, the supernatant was removed and neurospheres were mechanically dissociated using a P200 micropipette. Then, viable cells were counted, and 20 × 10^3^ cells were plated onto pre-coated poly-d-lysine/laminin (PDL/L) (100 g/mL) (Sigma-Aldrich) flat 96 multi-well microscopy plates (Cat.No:89626, IBIDI, Gräfelfing, Munich, Germany) and immediately treated with either regular ASCs secretome (SEC), DHA-treated ASCs secretome (ω-SEC) and respective positive or negative controls. Positive control media refers to Complete NeuroCult™ media while negative control media refers to Neurobasal A medium supplemented with 1% Glutamax (Gibco) and 1% kanamycin (Life Technologies). The plates were placed in an incubator at 37 °C, 5% CO2, 95% air and 90% relative humidity for 5 days.

## Neurodifferentiation and neurite outgrowth of hNPCs

To assess neurodifferentiation, hNPCs were probed using a double immunofluorescence strategy with antibodies against proteins present in immature (AB18723, Abcam, Cambridge, Cambridgeshire, UK—rabbit anti-Doublecortin 1:300) or mature neurons (M4403, Sigma—mouse anti-MAP2 1:500). Briefly, at the end of day 5, cells were washed once with PBS, fixed with 4% PFA for 15 min and washed again with PBS. Subsequently, cells were blocked using PBS 0.2% Triton X-100 (0.2% PBS-T) with 10% newborn calf serum (NBCS, ThermoFisher Scientific) at RT° for 1 h, followed by incubation with the primary antibody mixture in the same buffer but with 4% NBCS for 4 h at RT°. Following three washes with PBS, primary antibodies were detected with secondary antibodies (Alexa Fluor 488 goat anti-mouse (1:1000, Invitrogen) and Alexa Fluor 594 goat anti-rabbit (1:1000, Invitrogen) incubated in the same buffer as the primary antibodies for 2 h at RT°. DAPI counter staining was performed for 10 min at RT°, followed by 2 additional PBS washes. Images were acquired in an IX81 inverted fluorescence microscope (Olympus) using a 4 × objective (UPLANFL NA 0.13) for whole-plate high speed scanning of total field of view (FOV) and a 20 × objective (LUCPLFLN NA 0.45), for multiple single point image acquisition. Acquisition parameters between groups were kept constant across the three individual experiments. A total of 10 images per well from 5–11 wells per group were acquired and analyzed using purpose-built macros in Fiji. Briefly, total cell numbers were quantified per FOV of 20 × images based on the threshold segmentation and processing of DAPI signal. Given the presence of doublecortin (DCX) and MAP2 signal in cytosolic regions, positive cells were quantified using an enlarged mask of the binary segmentation of the DAPI signal that was superimposed onto the DCX and MAP2 channels respectively using a transparent-white function. This allows for the automatic quantification of positive cells for either marker of interest in a large number of images. Quantification of neurite length was made using the line tool to manually segment the longest neurite of 10 neurons per image.

## Statistical analysis

A confidence interval of 95% was used for all statistical tests. The normality assumption was tested for all continuous variables through evaluation of the frequency distribution histogram, the values of skewness and kurtosis, and by the Shapiro–Wilk test. The assumption of homoscedasticity was tested by Levene’s test. An unpaired t-test was used to evaluate mean differences between two samples. The one-way ANOVA was used to evaluate mean differences between more than two samples with one independent variable. Multiple comparisons were performed with Tukey’s or Dunn’s post-hoc tests for pairwise comparison of the independent variable. Mixed design ANOVA with Tukey's or Dunn's post hoc test for pairwise comparison of the independent variable was used to evaluate mean differences in samples with one independent variable and one repeated measure. Results are expressed as mean ± SEM. All statistical analysis and graphing was performed using Prism GraphPad version 9.0.1 (GraphPad Software; La Jolla, USA) and results were considered significant at *p* ≤ 0.05. Effect sizes and exact P values are reported in Supplementary Table 2.

## Results

### Dose response analysis of DHA priming reveals a morphologic phenotype that correlates with increased metabolic viability and secretome protein output

**Fig. 1:**
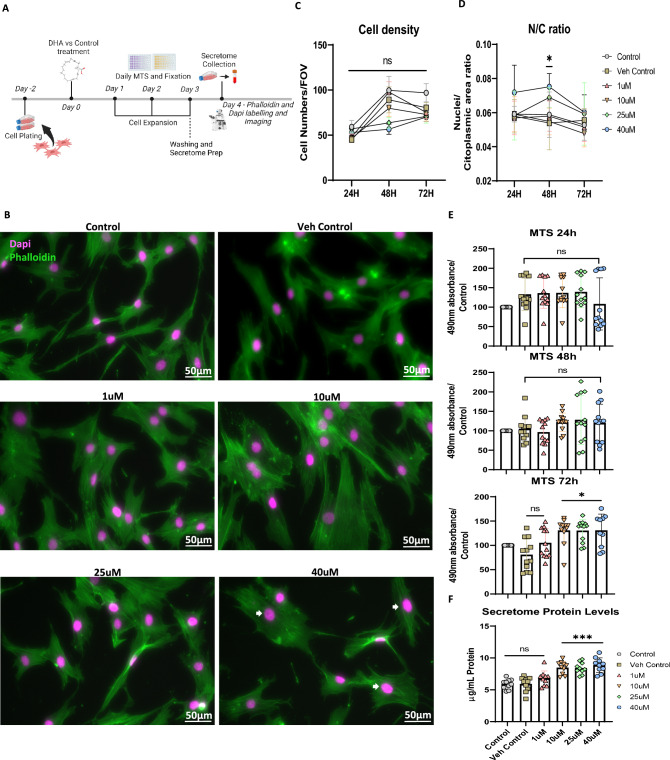
Temporal and concentration–response analysis of ASCs priming with DHA. Experimental outline for DHA priming with multiple concentrations and temporal assessment of metabolic viability and morphometric parameters as well as secretome collection (Created with BioRender.com) (**A**). Representative wide-field photomicrography of ASCs stained for Phalloidin and Dapi, pseudo-colored as green and magenta respectively. White arrows marks representative larger nuclei (**B**). Cell density analysis expressed as cell numbers per field of view of a 20 × image (**C**). Morphometric nuclei to cytoplasmic area ratio analysis shows a significant response at 48 h for the 25 and 40 µM DHA-treated ASCs (**D**). Analysis of metabolic viability expressed as the percentage of the control shows an increase in the metabolic viability for 10, 25 and 40 µM at 72 h of DHA treatment (**E**). Quantification of concentration–response effect on secretome total protein show 10, 25 and 40 µM to increase the amount of secreted protein in the secretome of ASCs when compared to control (**F**). Data shown as mean ± SEM. (n = 8 to 12/group for C), (n = 8/group for D), (n = 10 to 12/group for E) and (n = 10/group for F). *p < 0.05, ***p < 0.001, ns = non-significant

To assess the best concentration and exposure time to prime ASCs with DHA, cells were exposed to increasing concentrations ranging from 1 to 40 µM for either 24, 48 or 72 h (Fig. 1A). Given the importance of having adequate cell numbers for secretome production as shown in previous protocols, cell growth dynamics was analyzed over time [11]. As the secretome protein output is a major bottleneck for scaling-up translational strategies, a morphometric marker of biosynthetic capacity (nuclei to cytoplasm size ratio) was also analyzed [25]. Cell density results from microscopic evaluations showed that the different DHA concentrations did not significantly affect cell growth dynamics at the assessed time-points demonstrating a lack of cytotoxicity on ASCs (Fig. 1B and C). Morphometric analysis revealed that cells exposed to 40 µM DHA had an increase in the nuclei to cytoplasmic ratio (N/C ratio) at 48 h of exposure which returned back to control levels at 72 h. The cells also showed a small response to the 25 µM dose at 48 h, although not statistically significant. All the other concentration groups were similar to control, demonstrating a possible dose threshold for the response on this morphological adaptation (Fig. 1C, D and Supplementary Fig. 1). To evaluate if this effect could be coupled to metabolic viability for secretome production, we assessed the metabolic viability response throughout time in combination with measurements of the total protein produced in the secretome of ASCs. Metabolic viability was increased in cells treated with DHA 10, 25 and 40 µM at 72 h when compared to control, which was associated with an increased total protein output in the secretome of ASCs (Fig. 1E and F). To our knowledge, this magnitude of increase in total secretome protein content (± 50%) is a non-trivial response that has not yet been reported with any other molecular priming strategy. For instance, hypoxia preconditioning of ASCs with a 48 h secretome production time yielded a (± 30%) increase over the control [26]. Overall, the phenotypic changes observed after priming demonstrated that the highest concentrations of 25 and 40 µM performed the best. However, taking a collective consideration of the data, cells treated with 40 µM had an advantage over the 25 µM group in the degree of phenotypic adaptations prompting us to use this concentration for further studies.

### DHA treatment maintains the expression of phenotypic markers and increases the differentiation capacity of ASCs towards the adipogenic lineage

**Fig. 2:**
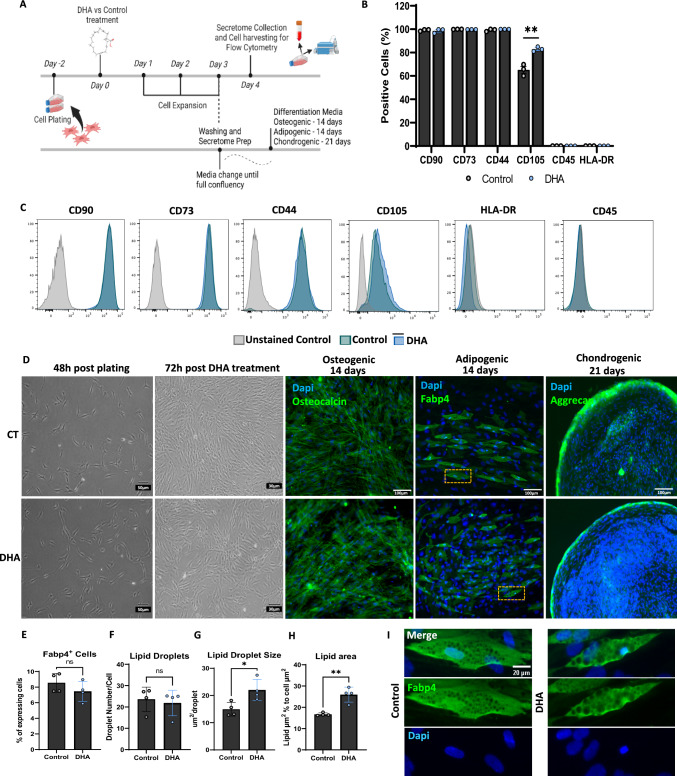
The effects of DHA priming on phenotypic and multi-lineage differentiation potential of ASCs. Experimental outline for DHA priming, harvesting of cells for flow cytometry and treatment of confluent cells with differentiation medias (Created with BioRender.com) (**A**). Flow cytometry quantification of the percentage of cell expressing phenotypic markers (CD90, CD73, CD44, CD105, CD45 and HLA-DR) (**B**). Representative flow cytometry histograms of Control (green) and DHA-treated (blue) in comparison to Unstained Control (grey) (**C**). Representative bright-field and fluorescence wide-field microscopy images of cells at the DHA treatment time-point, 72 h after treatment and after each differentiation protocol was completed (**D**). Quantification of number of FABP4^+^ cells expressed as a percentage of total cells (**E**). Quantification of the mean number of lipid droplets per FABP4^+^ cell (**F**). Quantification of lipid droplet size expressed as squared micrometers per droplet (**G**). Quantification of the area occupied by lipid droplets within each FABP4^+^ cell normalized by cell size (**H**). Representative photomicrograph of FABP4^+^ cells of yellow insets from D depicting bigger lipid droplets that occupy a larger cell area fraction (**I**). Data shown as mean ± SEM. (n = 3/group for B and C), (n = 4/group for D to I). *p < 0.05, **p < 0.01, ns = non-significant

To ensure that ASCs still retained their phenotype after priming, cells were exposed to DHA at 40 µM using the same expansion protocol, followed by conditioning for secretome collection (Fig. 2A). The cells that produced the secretome were collected and stained for established MSC identity markers. Markers expressed by MSCs include CD90, CD73, CD44, and CD105, while CD45 and HLA-DR are absent in these cells as represented in the gating strategy in Supplementary Fig. 2 [3, 4]. Flow cytometry results demonstrated that DHA treatment maintained the overall phenotypical identity of MSCs for most markers except CD105 (Fig. 2B and C). The lower percentage of cells expressing CD105 found in both conditions could be related to the 24 h culture in serum-free media (during secretome production), as it has been shown previously [27]. Nevertheless, the percentage of CD105^+^ cells was increased with DHA treatment when compared to control. This effect may represent an adaptation towards the adipogenic lineage as CD105 has been demonstrated to interact with important ligands of the TGF-β/SMAD signaling pathway, shown to be relevant for adipogenic induction [28]. When investigating the multi-lineage differentiation potential, DHA priming was shown to have no negative consequences. As shown in (Fig. 2D), both control and DHA treated cells were able to differentiate along the osteogenic, adipogenic and chondrogenic lineages after reaching confluence when treated with standard differentiation cocktails. Differentiation along the osteogenic and chondrogenic lineages presented prototypic characteristics of cobblestone-like osteoblasts and proteoglycan accumulation respectively, without marked differences between groups. Since DHA treatment had a mild but significant effect on CD105 expression and given the relationship to adipogenic induction, we sought to define whether DHA priming had a specific impact on the adipogenic phenotype. Analysis of fatty acid binding protein 4 (FABP4) staining showed marked effects on the adipogenic phenotype. Although the total number of FABP4^+^ cells as well as the total number of lipid droplets per cell were similar after DHA priming (Fig. 2E and F), the size of individual lipid droplets and the total area they occupied within each adipocyte were significantly increased (Fig. 2G–I). These results support the idea that DHA induces specific adipogenic transcriptional programs that control the behavior of adipose stem and progenitor cells [19].

### Priming with DHA increased population doubling time and modulated ASCs functional and transcriptional responses to replicative senescence induction

**Fig. 3:**
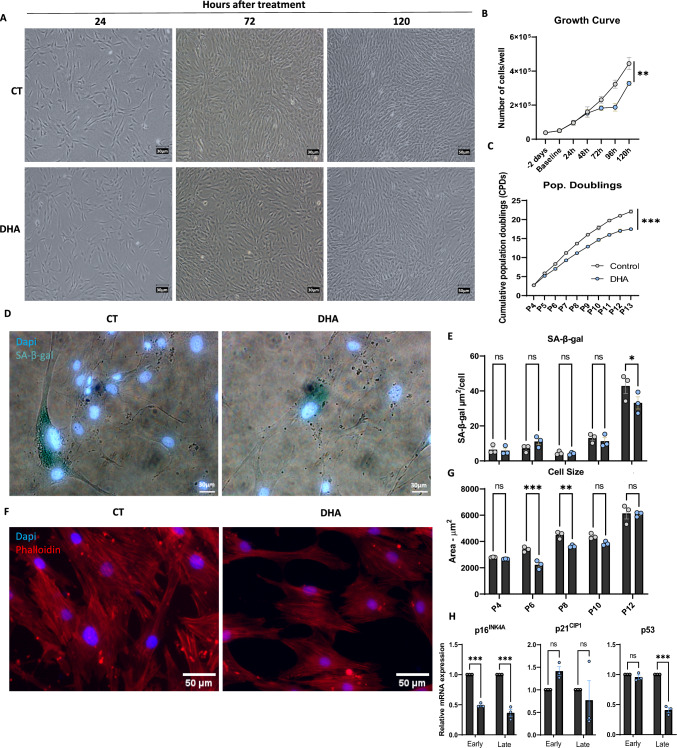
Probing the effects of DHA priming on the proliferative rate and senescence phenotype of ASCs. Representative bright-field photomicrographs obtained every 48 h along the ASCs growth curve experiments (**A**). Growth kinetics of ASCs at passage 6 in the secretome collection paradigm (**B**). Quantification of the cumulative number of duplications throughout multiple passages (**C**). Representative merged bright-field and fluorescence wide-field microphotographs of ASCs stained for DAPI (blue) and SA-β-gal (dark green) (**D**). Quantification of SA-β-gal staining throughout multiple passages (**E**). Representative wide-field fluorescence microscopy of ASCs at P6 stained for Dapi and Phalloidin for the morphometric analysis over multiple passages (**F**). Quantification of cell size over multiple passages (**G**). Gene expression analysis of senescence associated genes (**H**). Data shown as mean ± SEM. (n = 6 to 9/group for B), (n = 3/group for C, E, G and H). *p < 0.05, **p < 0.01, ***p < 0.001, ns = non-significant

Since the production of cell secretome for therapeutic applications requires high cellular yields and sometimes multiple passages, we asked whether DHA had an impact on ASCs proliferation in two distinct growth paradigms (Supplementary Fig. 3A). The first was aimed at studying the impact of DHA priming on the secretome collection paradigm of P6 cells involving 48 h of cell adhesion after plating, followed by 72 h of DHA treatment plus 24 h of secretome conditioning in serum free and DHA free media. An extra 24 h time-point (120 h) was added to observe if a ceiling effect on proliferation could be observed (Fig. 3A). Data showed that DHA-treated cells grew at a similar rate to control until 72 h, slowing their proliferation at both 96 and 120 h. Importantly, by observing the growth curves and the relative difference to control, it is possible to notice a recovery of proliferative potential at the last time point (Fig. 3B). This result could indicate a possible metabolic change of the DHA treated cells, because after the media change (72 h time-point) cells proliferative rate returns as seen by the slope of the curve. The second growth paradigm was aimed at assessing the impact of DHA treatment on the long-term expansion of ASCs until the point of replicative senescence induction. Quantification of cumulative population doublings confirmed that overtime, DHA treated cells proliferated slower (Fig. 3C). Importantly, additional analysis ruled out the possibility of this result being caused by either reduced adhesion or increased apoptosis induction in DHA treated cells (Supplementary Fig. 2B–F). The temporal functional analysis of senescence induction revealed that DHA-treated cells exhibited reduced SA-β-gal staining at P12 which correlated with reduced cell sizes at earlier passages (P6 and P8) (Fig. 3D–G). These results were also associated with reduced expression of the master regulator of senescence induction p16^INK4A^ at both P6 and P12 passages as well as reduced expression of p53 at P12 (Fig. 3H). Collectively, this data indicates a differential response of DHA-treated ASCs to the process of replicative senescence induction which may have implications for their paracrine activity.

### Priming ASCs with DHA induces changes in the neuroregulatory profile of the secretome leading to positive effects on neuronal differentiation and neuritogenic potential

**Fig. 4:**
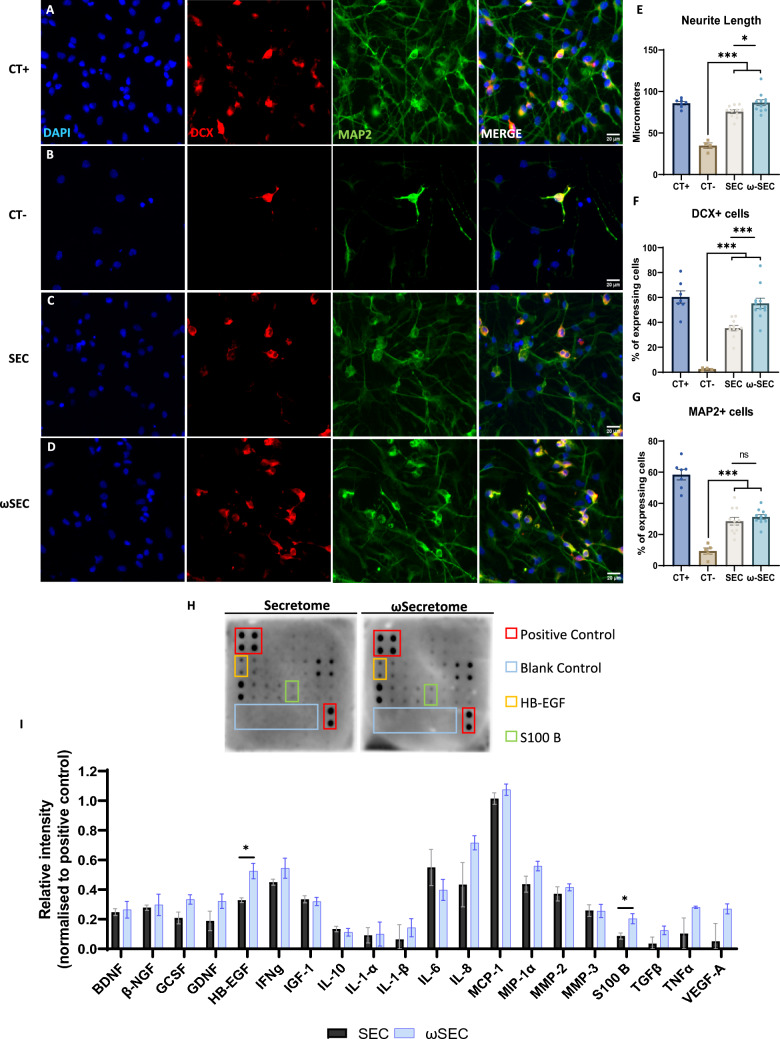
Differential response assessment of primed ASCs secretome in neurite outgrowth and neural differentiation potential of human neural progenitor cells. Representative merged and respective single-channel wide-field fluorescence photomicrographs of human NPCs stained for Dapi (Blue), Map2 (Green) and Doublecortin (Red) (**A**–**D**). Quantification of neurite length of hNPCs expressed as mean of longest neurite length (**E**). Quantification of Doublecortin expressing cells shown as a percentage of total cells (**F**). Quantification of MAP2 expressing cells shown as a percentage of total cells (**G**). Representative multiplex membrane-based antibody array for detection of neuroregulatory proteins in the secretome of ASCs, demonstrating controls and significantly altered proteins (**H**). Quantification of neuroregulatory proteins based on image densitometry analysis (**H**). Data shown as mean ± SEM. (n = 4 to 11/group for E and F), (n = 5 to 11/group for G) and (n = 4/group for H and I). *p < 0.05, ***p < 0.001, ns = non-significant

To functionally test the impacts of DHA priming on the neuroregulatory profile of ASCs secretome, an established human neural progenitor cell (hNPCs) differentiation assay was employed [16, 29]. NPCs grown as neurospheres were dissociated and allowed to differentiate while incubated with ω-secretome (ω-SEC) or control secretome (SEC) for 5 days. The analysis of neural differentiation markers showed that secretome-treated cells were able to survive and extend their MAP2-labelled processes to a length similar to the Control^+^ condition (Fig. 4A–E). Moreover, this neuritogenic effect was significantly higher in ω-SEC treated NPCs when compared to SEC. When assessing the neurodifferentiation potential of both secretomes, they were able to support the survival of early Doublecortin (DCX) positive^+^ neuroblasts when compared to Control^−^, with ω-SEC significantly increasing the proportion of cells retaining this progenitor phenotype when compared to SEC (Fig. 4F). Additionally, secretomes were able to support neuronal maturation as evidenced by the increased proportion of MAP2 positive^+^ cells when compared to Control^−^, irrespective of priming (Fig. 4G). To gain mechanistic insight into these differences between ω-SEC over SEC, equal amounts of secretomes (controlled for protein level) were tested using a membrane-antibody array assessing the expression of 20 neuroregulatory proteins. The results showed that overall, the expression of neurotrophic factors, cytokines and chemokines was similar between secretomes with a tendency to a higher concentration in the ω-SEC group (Supplementary Fig. 4). Importantly, the expression of heparin binding epidermal growth factor-like growth factor (HB-EGF) and S100B, two factors with neurotrophic properties, was significantly higher in ω-SEC when compared to SEC. Overall, these data support the notion that molecular priming of ASCs with DHA promotes the production of secretome with enhanced neuroregulatory potential.

## Discussion

Several studies have demonstrated the feasibility of priming strategies to optimize MSC’s for therapeutic applications. While some strategies have focused on the manipulation of cell culture conditions, such as the use of biomaterials [22], hypoxia [15] or bioreactor cultures [16, 30], the majority has used inflammation-modulating mediators to induce beneficial phenotypic changes in MSCs [14]. In this study we employed for the first time a priming strategy inspired by the biology of adipose-tissue to improve the neuroregulatory capacity of the ASCs secretome. It has been demonstrated that adipose progenitors are positioned in close proximity to the adipose tissue vasculature, where they are able to sense the cues of the vascular microenvironment [17]. Specifically, these cues are sensed by the primary cilium through the presence of free fatty-acid receptors (FFARs) [19]. DHA, a natural ligand of the ciliary FFAR4, has been shown to induce favorable adaptations in adipose progenitor cells phenotype, leading to adipogenic transcriptional changes [19]. Therefore, we developed a DHA priming strategy in ASCs and studied its effects on several aspects of ASCs biology and the neuroregulatory function of its secretome.

The initial experiments, conducted to define the optimal DHA concentration for priming, showed that the highest concentration tested (40 µM), induced more pronounced phenotypic changes. DHA priming led to cells exhibiting increased metabolic activity coupled with higher biosynthetic capacity as determined by a morpho-functional shift and higher amount of protein levels in the secretome. Together, these phenotypic changes induced by DHA may be linked to enhanced stem cell function [31]. Evidence shows that cell growth and division are only loosely coupled, marked by expansion in cell size when the kinetics of cell-cycle progression is reduced [32]. In our study, DHA inverted this relationship, as cells tended to proliferate at a slower rate but presented an increased N/C ratio. These findings may have important implications for secretome therapeutic applications in the CNS. For instance, if the higher protein yield in the primed secretome is the result of increased growth factor concentration, a higher bioactive response could be expected due to the increased availability of proteins with therapeutic potential reaching the brain. Considering this, a large-scale analysis studying more than twelve cell sources of therapeutic extracellular vesicles identified, dose as a crucial determining factor of bioactivity [33]. However, it is worth noting, that increased concentrations of growth factors or other therapeutic agents may not linearly correlate with enhanced bioactivity in all disease contexts and dose-escalation assays should be in-built in translational pipelines exploring cell secretome therapeutically. Accordingly, after correcting for total protein content, DHA priming increased the levels of 17 out of 20 proteins with known neuroregulatory functions, with HB-EGF and S100B being significantly elevated 1.6 and 2.3 fold, respectively, over controls. The mechanisms underlying these effects are likely to result from complex interactions between anabolic and catabolic pathways. For instance, several lines of evidence show that DHA activates pathways responsible for protein synthesis such as the mTOR pathway in human tissues [34]. Another possible mechanism to explain the increased protein secretion by DHA-treated ASCs is increased exocytosis. Indeed, recent investigations suggest that DHA regulates exocytotic fusion pore dilation and facilitates the fusion of exocytotic vesicles [35, 36]. Collectively, these mechanisms provide a rationale for the pleiotropic functions of DHA in modulating secretome production of ASCs.

Concerning the effect of DHA priming on the phenotype identity of ASCs, the data showed that DHA maintained the overall characteristics of MSCs such as expression of CD73, CD90 and CD44 and absence of CD45 and HLA-DR expression. DHA treatment induced a small but significant increase in the expression of CD105, which could have led to adipogenic pre-adaptation given the involvement of CD105 in the functioning of the TGF-β/SMAD signaling axis [37]. CD105 has two isoforms, a long and a short variant, with opposing roles in TGF-β signaling, with the long version inhibiting TGF-β mediated SMAD3 activity while increasing SMAD1, which is necessary for adipogenesis of both white and brown adipocytes [28]. Consistent with this, our adipogenic differentiation data show that, DHA pre-treatment resulted in a substantial increase in individual droplet size and area occupancy within differentiated adipocytes. Although we could not establish a causal relationship with CD105 expression and adipogenesis, this finding is in line with recent research showing that DHA is a potent adipogenic inducer, even being able to compensate for the titration in components of the standard adipogenic media such as insulin or dexamethasone [19]. The effects of DHA priming on the osteogenic and chondrogenic differentiation were shown to be minor, and overall did not alter differentiation trajectories along these lineages.

Our analysis of the impact of DHA priming of ASCs in two distinct proliferation paradigms showed that DHA increased cell doubling time both acutely and throughout treatment over multiple passages. This finding aligns with the studies examining DHA as a potential strategy to reduce cancer cell proliferation [38]. Given the relative similarity between cancer cells and cells with stem cell potential, these effects of DHA could be expected [39]. Although important, this confirmatory finding served mostly to describe the effects of DHA on the *in vitro* proliferation of ASCs. What was important to demonstrate was that cells under DHA expansion were still able to proliferate and generate yields that are more than sufficient for secretome production for translational applications. In the long term expansion paradigm, both DHA-treated and control cells decreased their proliferative capacity, and increased their cell area and SA-β-gal expression (a prototypical replicative senescence response). However, chronic treatment with DHA led to an earlier onset reduction in proliferative capacity. Nevertheless, when comparing cells in their last passage, chronic DHA exposure led to reduced SA-β-gal area coupled with reduced p16^INK4A^ and p53 gene expression highlighting a function for DHA in modulating the acquisition of senescent phenotype after replicative pressure. Furthermore, DHA treatment at the passage of secretome collection (P6), did not alter the senescence phenotype as seen by the early gene expression, SA-β-gal as well as the expression of senescence associated secretory profile (SASP) proteins, such as IL6, IL8 and MCP1. Caution should be taken to interpret these data, as DHA treated cells have a longer doubling time, a reduced rate of telomere attrition is expected throughout passages which would explain the reduction in SA-β-gal expression at the latest passages [40]. In addition, it is important to remark that our paradigm did not allow for the comparison of SA-β-gal labeling in proliferation arrested cells from the control group, as these cells still presented some residual proliferative capacity at P12. The mechanisms underlying these effects are likely to be complex and multifactorial as they may derive from both activation of lipid receptors and/or metabolization of DHA after incorporation in membrane phospholipids. For instance, engagement of nuclear receptors such as peroxisome proliferator-activated receptor gamma (PPARγ) has been shown to induce p21^CIP1^ expression [41]. In accordance, DHA is a well-known activator of these receptors, potentially explaining why we detected a higher expression of p21^CIP1^ at the early passage, although not statistically significant [42].

Available evidence also suggests that these effects could be derived from changes in the lipid composition of cell membranes rather than through activation of lipid receptors. In fact, it has been demonstrated that bone-marrow MSCs from old donor’s (mean age 74.6 years-old) present drastic changes in membrane lipid compositions compared to MSCs from young donors (mean age 22.2 years-old). Specifically, this alteration is reflected by an increase in the concentration of arachidonic acid (ARA) to the detriment of DHA [43]. Additionally, a DHA treatment strategy similar to the one used in this study was shown to increase the concentration of plasma membrane DHA in bone-marrow isolated MSCs [44]. As both ARA and DHA can be metabolized by oxidizing enzymes to generate distinct bioactive oxylipins, it is expected that the different classes of oxylipins generated could mechanistically mediate the effects of DHA on senescence. In this context, the production of prostaglandin E2 (PGE2) by cyclooxygenase 2 (COX2) metabolism of ARA has been shown to be a hallmark of senescence and is part of the senescence associated secretory phenotype (SASP) [45]. Taken together, given our long-term exposure of ASCs with DHA, an enrichment of membrane DHA is expected. Therefore, a reduction of ARA-derived oxylipins from DHA enriched cell membranes may have halted the long-term adaptations related to replicative senescence induction.

Considering that the functional effects associated with MSC therapy are linked to their secretome activity, we tested as a proof-of concept, the possibility of DHA to modulate the neuroregulatory potential of ASCs secretome using a human neural differentiation assay. Our data demonstrated that both secretomes were able to support the survival, differentiation and neurite outgrowth of hNPCs. This finding is consistent with previous studies that show that ASCs secretome can induce hNPC differentiation as well as neurite outgrowth from dorsal root ganglia explants *in vitro* [46]. Importantly, ω-SEC induced functionally enhanced responses promoting neurite outgrowth as well as maintaining a larger progenitor cell pool marked by higher percentage of DCX^+^ cells when compared to regular SEC. These effects can be directly associated to the higher expression of specific neurotrophic factors found in the ω-SEC. It is important to note that, for total protein matched secretomes, all of the probed neurotrophins tended to be higher in ω-SEC when compared to the control SEC. Although some immunomodulatory proteins such as IL8, IL6 and TGFβ were found to be differentially expressed between ω-SEC and SEC, their differences were not statistically significant. This prompted us to focus the discussion specifically on neurotrophins. Indeed, heparin-binding EGF-like growth factor (HB-EGF) was significantly increased in ω-SEC when compared to SEC. Available evidence suggests that HB-EGF has pleiotropic effects on both neurite extension and neural differentiation which are mediated by activation of EGFR and ERB4 receptors. Results from, *in vivo* and *in vitro* studies demonstrate the effects of HB-EGF to be more restricted to the pool of early neuroblasts, where it supports survival, proliferation and migration through activation of EGFR [47, 48]. Notably, the effects of HB-EGF on mature neurons are thought to derive from ERB4 activity [48]. Another factor with neurotrophic properties found to be increased in ω-SEC was S100B. The neurotrophic effects of S100B have been demonstrated in multiple neuronal *in vitro* culture systems, being supportive of neurite extension and neuronal survival. The mechanism of action has been shown to be dependent on the activation of the receptor for advanced glycation end products (RAGE) [49]. Importantly, RAGE activation by S100B was also shown to be relevant for cell differentiation, specifically in inducing expansion of myoblast progenitors after acute muscle injury [50]. This finding aligns with our ω-SEC results on the DCX^+^ hNPC population supporting the possible role of S100B to support neural differentiation via the maintenance of early neuroblasts. Taken together, ω-SEC’s differential effects on enhanced neurite outgrowth and increased neural progenitor cell pool maintenance are likely related to its improved neurotrophic profile.

In summary, this study proposes a novel molecular priming strategy using DHA to drive cellular adaptations and improve the neuroregulatory potential of ASC secretome. The priming strategy presented here increased the amount of protein produced and significantly enhanced the levels of two neurotrophic factors (HB-EGF and S100B) in the ASCs secretome. These findings have relevance for strategies aiming to employ cell secretome as a potential therapy for CNS disorders, as approaches that induce functional responses related to early development are currently at the forefront of CNS regenerative strategies. Although the mechanism through which DHA induced these phenotypic changes was not investigated, the observed functional effects witnessed with priming were largely centered on processes related to metabolic and stress responses. Future investigations should be focused on further elucidating the metabolic impact of DHA priming coupled with assessments of the transcriptional state as well as the downstream implications on the secretome proteome, lipidome and metabolome.

## Supplementary Information

Below is the link to the electronic supplementary material.

Supplementary file1 (DOCX 720 KB)

## Data Availability

Datasets are available upon reasonable request from the corresponding author.
