# Supplementary material for: Lipid Priming of Adipose Mesenchymal Stromal Cells with Docosahexaenoic Acid: Impact on Cell Differentiation, Senescence and the Secretome Neuroregulatory Profile
Source: Tissue Eng Regen Med. 2024 Nov 4;22(1):113–28. doi: 10.1007/s13770-024-00679-5 (PMC11711600; doi:10.1007/s13770-024-00679-5)
Supplement: Supplementary file 1 — Supplementary file1 (DOCX 720 KB) [file 13770_2024_679_MOESM1_ESM.docx]

**Supplementary Information – Tissue Engineering and Regenerative Medicine**

**Lipid priming of human adipose mesenchymal stromal cells with docosahexaenoic acid: Impact on cell differentiation, senescence and the secretome neuroregulatory profile**

**Jonas Campos^1,2^, Belém Sampaio-Marques^1,2^, Diogo Santos^1,2^, Sandra Barata-Antunes^1,2^, Miguel Ribeiro^1,2^, Sofia C. Serra^1,2^, Tiffany S. Pinho^1,2^, João Canto-Gomes^1,2^, Ana Marote^1,2^, Margarida Cortez^1,2^, Nuno A. Silva^1,2^ Adina T. Michael-Titus^3^, António J. Salgado^1,2*^**

**1 Life and Health Sciences Research Institute (ICVS), School of Medicine, University of Minho, 4710-057 Braga, Portugal**

**2 ICVS/3B’s – PT Government Associate Laboratory, Braga/Guimarães, Portugal**

**3 Centre for Neuroscience, Surgery and Trauma, The Blizard Institute, Barts and The London School of Medicine and Dentistry, Queen Mary University of London, London, E1 2AT, UK**

***Corresponding Author:** António J. Salgado – [asalgado@med.uminho.pt](mailto:asalgado@med.uminho.pt)


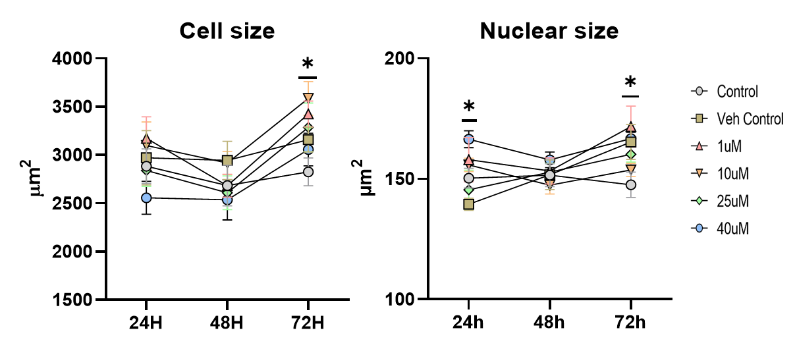
**Supplementary Figure 1**. Temporal analysis of morphometric parameters after exposure of A-MSCs to increasing concentrations of DHA. Data shown as mean ± SEM. (n=8 to 12/group for cell size) and (n=8/group for nuclear size), *P<0.05.


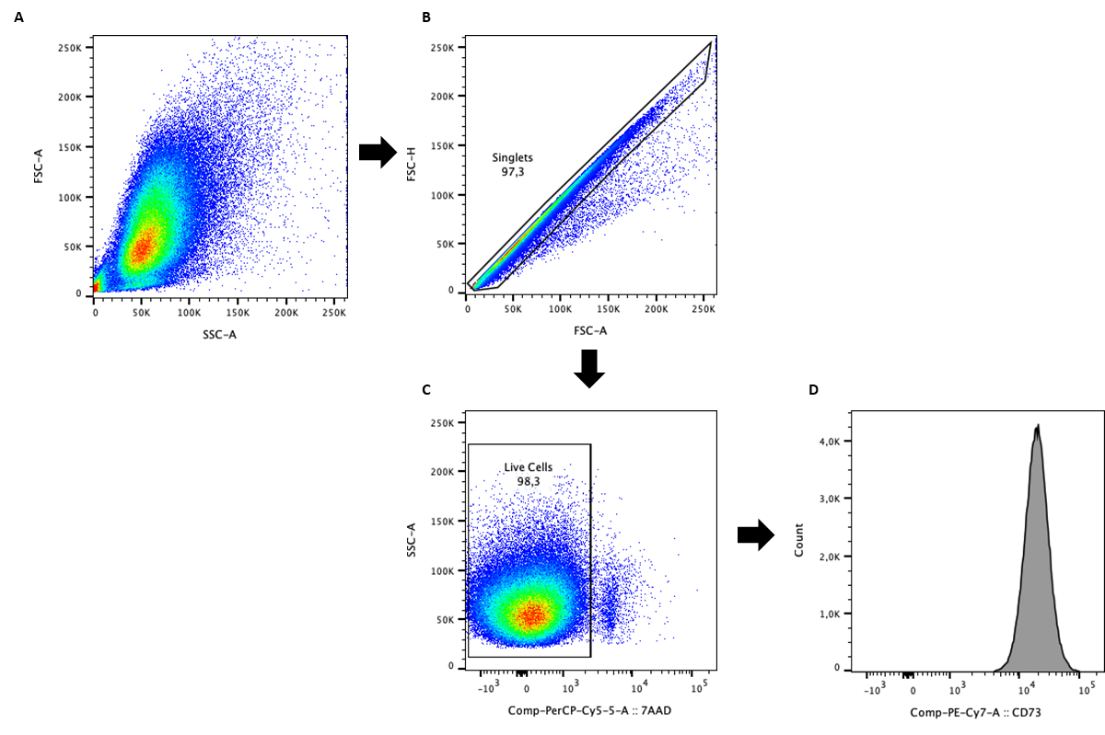
**Supplementary Figure 2**. Gating strategy for phenotypic characterization of mesenchymal stem cells (MSCs). Singlets were identified based on the area and height of the forward scatter (FSC) (A). Viable cells were selected by excluding 7-AAD positive cells (B and C). MSC lineage markers were then assessed through histograms (D), confirming the presence of CD90, CD73, CD105, CD44 and the absence of CD45 and HLA-DR. Example histogram provided of cells expressing CD73.

**Supplementary Figure 3**. Experimental outline for DHA priming and cell growth for the secretome collection (Fig 3.B) and for the replicative senescence (Fig 3.C) paradigms, as well as for SA-β-gal staining (Fig 3.E and G) and senescence associated gene expression analysis (Fig 3.H) (Created with BioRender.com) (A). Representative imaging pipeline and quantification of adhered cells (B and C). Representative immunofluorescence photomicrographs of cleaved-caspase-3 (ab3623, Milipore, 1:100) and acetylated tubulin (T6793, Sigma, 1:100) immunofluorescence (D). Quantification of nuclear versus cytosolic localization of cleaved-caspase-3 in A-MSCs treated with DHA and imaged at 48 h post treatment (E and F). Data shown as mean±SEM. (n=9/group for C) and (n=4/group for E and F), *P<0.05, ns = non-significant.


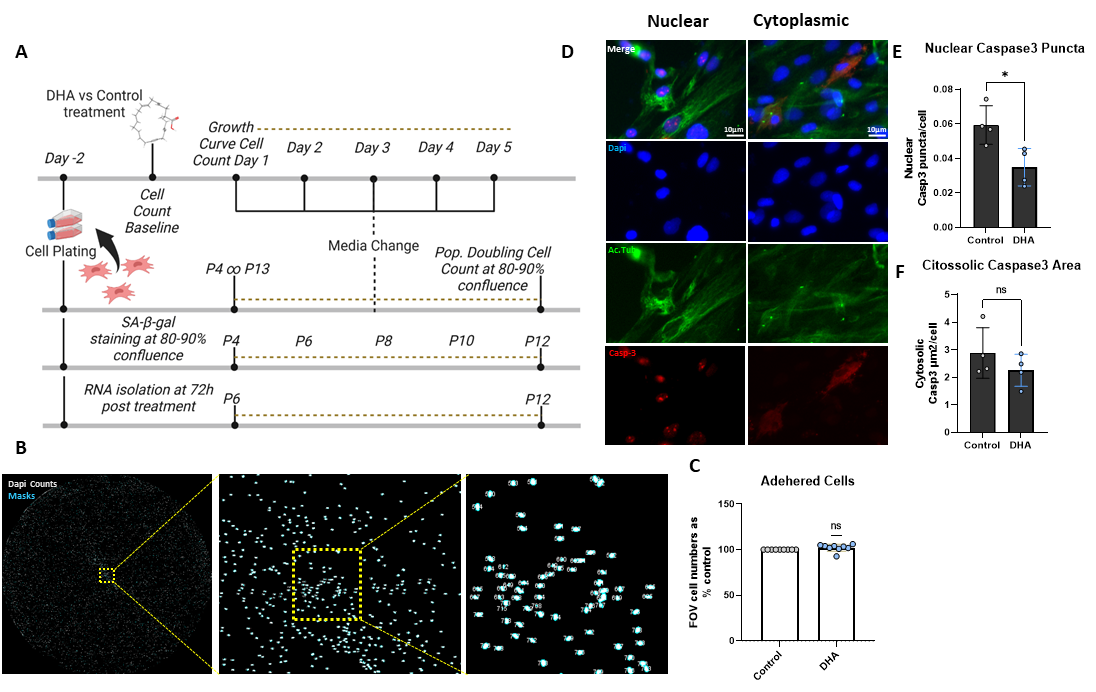


**
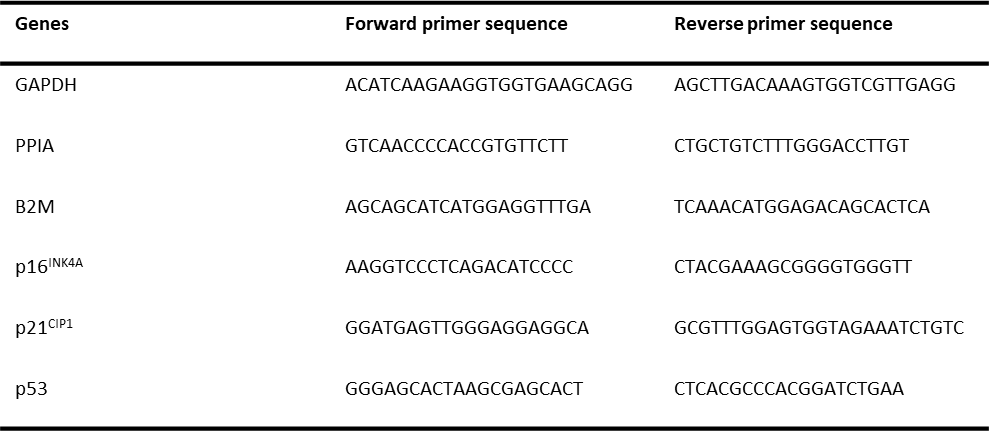
Supplementary Table 1.** Real-Time Quantitative PCR primer sequence information.

**Supplementary Table 2.** Statistical reporting.

| Figure | Statistical report | Sample size |
| --- | --- | --- |
| 1 D | *Two-way Anova*  *Time x Treatment Dosage Effect: F (10, 84) = 0.8818, P = 0.5534, η2p = 0.0950031*  *Time Effect: F (1.897, 79.66) = 5.563, P = 0.0063, η2p = 0.1169788*  *Treatment Dosage Effect: F (5, 42) = 7.600, P < 0.0001, η2p = 0.475* | 8 (per group) |
| 1 E | *One-way Anova -*  *F (5, 64) = 7.440, P<0.0001, Cohen's F=0.678233* | 10 to 12 (per group) |
| 1 F | *One-way Anova - F (5, 54) = 17.15, P<0.0001, Cohen's F = 1.1601006* | 10 (per group) |
| 2 B | *Unpaired T-test - t=5.784, df=4, P =* *0.0044, Cohen’s d = 4.723307* | 3 (per group) |
| 2 G | *Unpaired T-test - t=3.085, df=6, P =* *0.0215, Cohen’s d = 2.18257* | 4 (per group) |
| 2 H | *Unpaired T-test - t=5.179, df=6, P =* *0.0021, Cohen’s d = 3.662952* | 4 (per group) |
| 3 B | *Two-way Anova*  *Time x Treatment Effect: F (5, 67) = 8.251, P<0.0001, η2p = 0.3810909*  *Time Effect: F (5, 67) = 136.6, P<0.0001, η2p = 0.9106667*  *Treatment Effect: F (1, 16) = 6.403, P = 0.0223, η2p = 0.2858099* | 6 to 9 (per group) |
| 3 C | *Two-way Anova*  *Time x Treatment Effect: F (9, 36) = 21.91, P<0.0001, η2p = 0.8456195*  *Time Effect: F (9, 36) = 1333, P<0.0001, η2p = 0.9538533*  *Treatment Effect: F (1, 4) = 80.44, P=0.0009, η2p = 0.9526291* | 3 (per group) |
| 3 E | *Two-way Anova*  *Time x Treatment Effect: F (4, 16) = 2.712, P=0.0673, η2p = 0.4040524*  *Time Effect: F (4, 16) = 82.68, P<0.0001, η2p = 0.9970082*  *Treatment Effect: F (1, 4) = 1.391, P=0.3036, η2p = 0.2580226* | 3 (per group) |
| 3 G | *Two-way Anova*  *Time x Treatment Effect: F (4, 16) = 4.381, P=0.0140, η2p = 0.52273*  *Time Effect: F (4, 16) = 147.8, P<0.0001, η2p = 0.9736495*  *Treatment Effect: F (1, 4) = 18.12, P=0.0131, η2p = 0.8191682* | 3 (per group) |
| 3 H | ***P16*** *- Two-way Anova*  *Time x Treatment Effect: F (1, 4) = 3.969, P=0.1172, η2p = 0.498055*  *Time Effect: F (1, 4) = 3.969, P=0.1172, η2p = 0.498055*  *Treatment Effect: F (1, 4) = 317.3, P<0.0001, η2p = 0.9875506*  ***P53*** *- Two-way Anova*  *Time x Treatment Effect: F (1, 4) = 97.54, P=0.0006, η2p = 0.9606067*  *Time Effect: F (1, 4) = 97.54, P=0.0006, η2p = 0.9606067*  *Treatment Effect: F (1, 4) = 130.0, P=0.0003, η2p = 0.9701493* | 3 (per group) |
| 4 E | *One-way Anova -*  *F (3, 29) = 37.91, P<0.0001, Cohen's F=1.83179* | 4 to 11 (per group) |
| 4 F | *One-way Anova -*  *F (3, 29) = 31.13, P<0.0001, Cohen's F=1.6550199* | 4 to 11 (per group) |
| 4 G | *One-way Anova -*  *F (3, 30) = 52.01, P<0.0001, Cohen's F=2.1215283* | 5 to 11 (per group) |
| 4 I | ***HB-EGF*** *- Unpaired T-test - t=3.629, df=6, P =* *0.0110, Cohen’s d = 2.566358*  ***S100B*** - *Unpaired T-test - t=2.920, df=6, P =* *0.0266, Cohen’s d = 2.064681* | 4 (per group) |
| Supp. Fig 1  Cell Size  Nuclei Size | *Two-way Anova*  *Time x Treatment Dosage Effect: F (10, 84) = 1.865, P=0.0617, η2p = 0.1816853*  *Time Effect: F (1.915, 80.44) = 5.875, P=0.0047, η2p = 0.122702*  *Treatment Effect: F (5, 42) = 2.631, P=0.0371, η2p = 0.2385097*  *Two-way Anova*  *Time x Treatment Dosage Effect: F (10, 113) = 1.103, P = 0.3660, η2p = 0.0889301*  *Time Effect: F (1.957, 110.6) = 12.23, P<* *<0.0001, η2p = 0.1779037*  *Treatment Effect: F (5, 65) = 1.693, P=0.1488, η2p = 0.1152249* | 10 to 12 (per group)  8 (per group) |
| Supp. Fig 3  Nuclei Casp-3 | *Unpaired T-test - t=3.136, df=6, P =* *0.0202, Cohen’s d = 2.217687* | 4 (per group) |

**Supplementary Figure 4**. Volcano plot of identified proteins in the secretome of untreated and DHA-treated A-MSCs, showing increase in the expression of HB-EGF and S100B.

**Supplementary Table 3.** List of reagents and catalogs.

| **Reagent** | **Company** | **Catalog** |
| --- | --- | --- |
| **Cell culture reagents** |  |  |
| α-MEM | Invitrogen | 12000-063 |
| Neurobasal | Gibco | 21103-049 |
| NeuroCult | STEMCELL Techonologies | #05750 |
| FBS | Sigma-Aldrich | S0615 |
| PenStrep | Invitrogen | 15070-063 |
| Kanamycin | Gibco | 15160-047 |
| Trypsin | Gibco | 25300-062 |
| Adipogenic Media | STEMCELL Technologies | #05414  #05415 |
| Osteogenic Media | STEMCELL Technologies | #05467 |
| Chondrogenic Media | STEMCELL Technologies | #05457 |
| NBCS | ThermoFisher | 1610-159 |
| **Priming reagents** |  |  |
| DHA | Sigma-Aldrich | D2534 |
| FFA-BSA | Sigma-Aldrich | A8806 |
| **Dyes and antibodies** |  |  |
| Phalloidin | Sigma-Aldrich | P1951 |
| DAPI | Invitrogen | D1306 |
| Anti Osteocalcin | RD Systems | 967801 |
| Anti FABP4 | RD Systems | 967799 |
| Anti Aggrecan | RD Systems | 967800 |
| Anti CD90 | Biolegend | 328110 |
| Anti CD73 | Biolegend | 344010 |
| Anti CD105 | Biolegend | 323204 |
| Anti CD44 | Biolegend | 103001 |
| Anti CD45 | Biolegend | 304042 |
| Anti HLA-DR | Biolegend | 307646 |
| Anti Doublecortin | Abcam | AB18723 |
| Anti MAP2 | Milipore | M4403 |
| Goat- anti MS Alexa 488 | ThermoFisher | A11029 |
| Rabbit- anti GT Alexa 488 | ThermoFisher | A11078 |
| **Other Reagents** |  |  |
| MTS – Cell Titer 96 | Promega | G3581 |
| Bradford (Quick Start^TM^) | BioRad | #5000205 |
| SA-B-Gal kit | Abcam | Ab65351 |
| TripleXtractor | Grisp | #GB23.0100 |
| Xpert cDNA Supermix with gDNA eraser | Grisp | #GK86.0100 |
| XPert Fast SYBR mastermix | Grisp | #GE22.0100 |
| Human Neuro Discovery Array C1 | Ray Biotech | AAH-NEU-1–2 |
